# Augmenting apoptosis-mediated anticancer activity of lactoperoxidase and lactoferrin by nanocombination with copper and iron hybrid nanometals

**DOI:** 10.1038/s41598-022-17357-y

**Published:** 2022-08-01

**Authors:** Esmail M. El-Fakharany, Marwa M. Abu-Serie, Noha H. Habashy, Marwa Eltarahony

**Affiliations:** 1 Protein Research Department, Genetic Engineering and Biotechnology Research Institute (GEBRI), City of Scientific Research and Technological Applications (SRTA-City), New Borg El-Arab, 21934 Alexandria Egypt; 2 Medical Biotechnology Department, Genetic Engineering and Biotechnology Research Institute (GE-BRI), City of Scientific Research and Technological Applications (SRTA-City), New Borg El-Arab, 21934 Alexandria Egypt; 3 Biochemistry Department, Faculty of Science, Alexandria University, New Borg El-Arab, 21511 Alexandria Egypt; 4 Environmental Biotechnology Department, Genetic Engineering and Biotechnology Research Institute (GEBRI), City of Scientific Research and Technological Applications (SRTA-City), New Borg El-Arab City, 21934 Alexandria Egypt

**Keywords:** Biochemistry, Biotechnology, Cancer, Drug development

## Abstract

There is an urgent need in the medicinal fields to discover biocompatible nanoformulations with low cytotoxicity, which provide new strategies for promising therapies for several types of tumors. Bovine lactoperoxidase (LP) and lactoferrin (LF) have recently attracted attention in medicine for their antitumor activities with recognized safety pattern. Both LP and LF are suitable proteins to be coated or adsorbed to Cu and Fe nanometals for developing stable nanoformulations that boost immunity and strong anticancer effects. New nanometals of Cu and Fe NPs embedded in LP and LF forming novel nanocombinations of LP-CNPs and LF-FNPs had a spherical shape with an average nanosize of about 21 nm. The combination of LP-CNPs and LF-FNPs significantly exhibited the highest growth inhibitory efficacy, in terms of effectively lowering the half-maximal inhibitory concentration (IC_50_) values, against Caco-2, HepG2 and MCF7 cells comparing to nanometals, LP, LF and individual nanoproteins (LP-CNPs or LF-FNPs). The highest apoptotic effect of this nanocombination (LP-CNPs and LF-FNPs) was confirmed by the highest percentages of annexin-stained apoptotic cells and G0 population with the strongest alteration in the expression of two well-characterized apoptosis guards (p53 and Bcl-2) and the maximum suppression in the proliferation marker (Ki-67). Also, the in silico analysis predicted that LP-CNPs and LF-FNPs enhanced AMP-activated protein kinase (AMPK, p53 activator) activity and inhibited cancer migration-related proteases (cathepsin B and matrix metalloproteinase (MMP)-9). Our results offer for the first time that these novel nanocombinations of LP and LF were superior in their selectivity and apoptosis-mediating anticancer activity to Cu and Fe nanometals as well as the free form of these proteins or their individual nanoforms.

## Introduction

Lactoperoxidase (LP) and lactoferrin (LF) are glycoproteins with a molecular weight of about 80 and 78 kDa, respectively, that are well-known to play many biological and functional roles^1–3^. Despite whey-contained proteins being considered a minor part of bovine milk, it contains the most effective proteins and other components that exhibited a wide range of biological properties^4,5^. Both bovine LF and LP are considered the most effective constituents of whey proteins, besides other proteins such as α-lactalbumin (α-LA) and immunoglobulins. LF is one of the transferrin family, which plays an important role in releasing and chelating the iron from and to body cells, besides its contributing to many other relevant biological purposes such as antioxidant, antimicrobial, immunomodulatory and anti-inflammatory properties as well as protection against metastasis and tumor development^6,7^. In addition, many in vitro and in vivo studies demonstrated that the hololactoferrin (an iron saturated form of LF) and its derived peptides of LF have also been found to exert potent anticancer properties against many types of cancer cells^8–11^. However, LP is one of the peroxidases family, which contains a covalently linked heme prosthetic group in its catalytic center. The main function of peroxidases is primary control catalysis for the oxidation of certain exact components by using multiple stages to catalyze a related oxidation reaction in the existence of hydrogen peroxide (H_2_O_2_) and SCNˉ to produce many potent molecules with a varied antimicrobial and biological properties through a definite inhibitory system called LP system (LPS)^12–14^. LPS is not limited by the antimicrobial property but shows many other significant roles in the innate immunity system, degradation of some carcinogenic toxins such as aflatoxin and antioxidant effect^15–17^. Recently, nanoformulation of the therapeutic proteins provides a greater advance for scientific research and biotechnological applications to increase drug stability and efficacy over time. Thus have developed a great potential in various prospective, including cancer treatment and prevention, diagnosis, bioimaging and infectious diseases control.

Owing to the large surface-to-mass ratio and nano-size of nanoparticles (NPs), they can efficiently interact with therapeutic proteins as biological compounds forming NPs-protein corona (NP-PC). According to irreversible and revisable binding between protein and NPs, there are two types of corona, including hard corona or soft corona, respectively^18–20^. This NP-PC is a result of interaction between the protein and NPs or adsorption of the used protein on the surface of NPs. The adsorption of proteins on the surface of NPs is occurred as following many forces, including hydrogen bonds or Van der Waals interactions^21^. Nano-metals have been recognized for modification of their surface to achieve new applications such as their uses in dentistry and oral biofilm for infection management and control. The antibacterial mechanism mediated with the modified nano-metals may involve free metal ion toxicity and/or oxidative stress via the formation of activated oxygen species on the surface of NPs^14,22,23^. This generated activated oxygen species act mainly by attacking polyunsaturated phospholipids, which lead to damage of site-specific DNA^24,25^. In general, Ag, Au, Pd and Pt are the most extensively studied nano-metals for such binary systems^26,27^. The development of nanocomposite was boosted to enhance nano-metals properties for improving their functionalities and avoid the limitation of sole NPs^28^. However, the high cost, cytotoxicity and genotoxicity effects of these nanocomposites cause many limitations to their application in medical fields^29,30^. Thus, it becomes a great demand for substitution or modification of those metals with another alternative selected on the advantages, which have more safety, cost-effectiveness, reactivity, easy availability, catalytic features, and overall performance. Among these metals, iron (Fe) and copper (Cu) are naturally exist as micronutrients in human, animal and plant tissues; they play crucial roles in many biochemical functions such as cell wall metabolism, synthesis of the DNA, respiration and oxidative stress.

Herein, we aimed to investigate the increment in the antitumor effect of the novel nanofabricated LP and LF with both Fe NPs and Cu NPs. The Fe-based NPs gained full approval by the US Food and Drug Administration (FDA) for use in many applications, including treatment of iron deficiency anemia in adults with chronic kidney disease and magnetic resonance imaging applications in liver pathologies detection as well as in water purification approaches^29,31^. In the same perspective, the US Environmental Protection Agency (EPA) permitted the use of Cu-based NPs as an antimicrobial candidate^29^. Thus, both Fe and Cu NPs may offer a great promise to adsorb or interact with therapeutic proteins (LP and LF), which still have limitations in delivery at their standard pharmacodynamic parameters owing to their nature and instability which inhibits permeability and transport through plasma membrane^21,32^. Furthermore, adsorption and interaction of therapeutic proteins with nano-metals have been the subject of intense investigation and become the basis of NPs bio-reactivity^21^, which may lead to induce positive properties on the interactions and stability of the binding protein^33,34^.

In the light of the above, the present study focused on the nanofabrication of LP and LF with the biologically synthesized CuFe-based nanocomposites by actinomycetes isolate. The prepared nanocombinations of CNPs-LP and FNPs-LF were characterized morphologically and structurally. The activity of the LP and LF after nanofabrication was determined to prove the proteins are in suitable structure folding forms, which consequent non-changes in their activities. Consequently, the anticancer capability was determined against different types of cancer cell lines as well as the examination of their cytotoxic effect on the normal cells was performed. These in vitro anticancer effects were investigated by estimating the dose of growth inhibition, modifications in the treated cell morphology, cell cycle arresting, and percentage of apoptosis, as well as immunohistochemical and wound healing analysis. Detecting the expression levels of apoptosis-related genes in the tested breast cancer cell line was also included. Moreover, the predicted influence of the studied metalloproteins on the activity of 5′ adenosine monophosphate-activated protein kinase (AMPK, p53 activator), cathepsin B, and matrix metalloproteinase (MMP)-9 (cancer migration-related proteases) was evaluated. These novel modified nanocombinations might be useful in medicinal fields for controlling and treatment of cancer diseases.

## Results and discussions

### Nanofabrication, characterization and redox functions of LP-CNPs and LF-FNPs nanocombinations

The whey proteins are well-known as promising anticancer candidates, therefore there is an essential requirement for protecting them from the body barriers and improving their stability and delivery to target tumor sites as well as enhance their bioactive functions through nanofabrications^35^ with NPs such as nanometals. Both LP and LF are glycoproteins with a monomeric heme-containing bound calcium- and iron for LP and with non-heme bound iron for LF^35,36^. Both bovine LP and LF are highly cationic proteins with isoelectric point (*pI*) determined to be 9.6 and 9.5, respectively, and this high cationicity is one of the major important characteristics of their structures that make both of them have significant biological properties including antimicrobial efficacy and immunomodulatory roles^36–39^. Interaction or adsorption of protein on the surface of NPs leads to the formation of new complexes of NPs-protein, which is considered the origin of NPs bio-reactivity. The formed new complexes can change the structural conformation of the adsorbed proteins on the surface of NPs, which lead to modifying the total bio-reactivity of the formed NPs. Furthermore, the surface of NPs can affect and modify the structure of the adsorbed protein, which leads to a change in the main function of the protein^21^. In the present study, both bovine LP and LF were purified from skim milk by using a Mono S column as a cation exchange chromatography column through using a NaCl gradient. All fractions containing LP or LF were collected separately, dialyzed, concentrated, and applied into the Sephacryl S200 column for further fractionation^40^. For the first time, the purified LP and LF were used to prepare nanoforms of LP and LF with Cu and Fe biologically synthesized nanocomposites (CNPs and FNPs, respectively) by *S. cyaneofuscatus* EM3 strain^41^.

**Figure 1:**
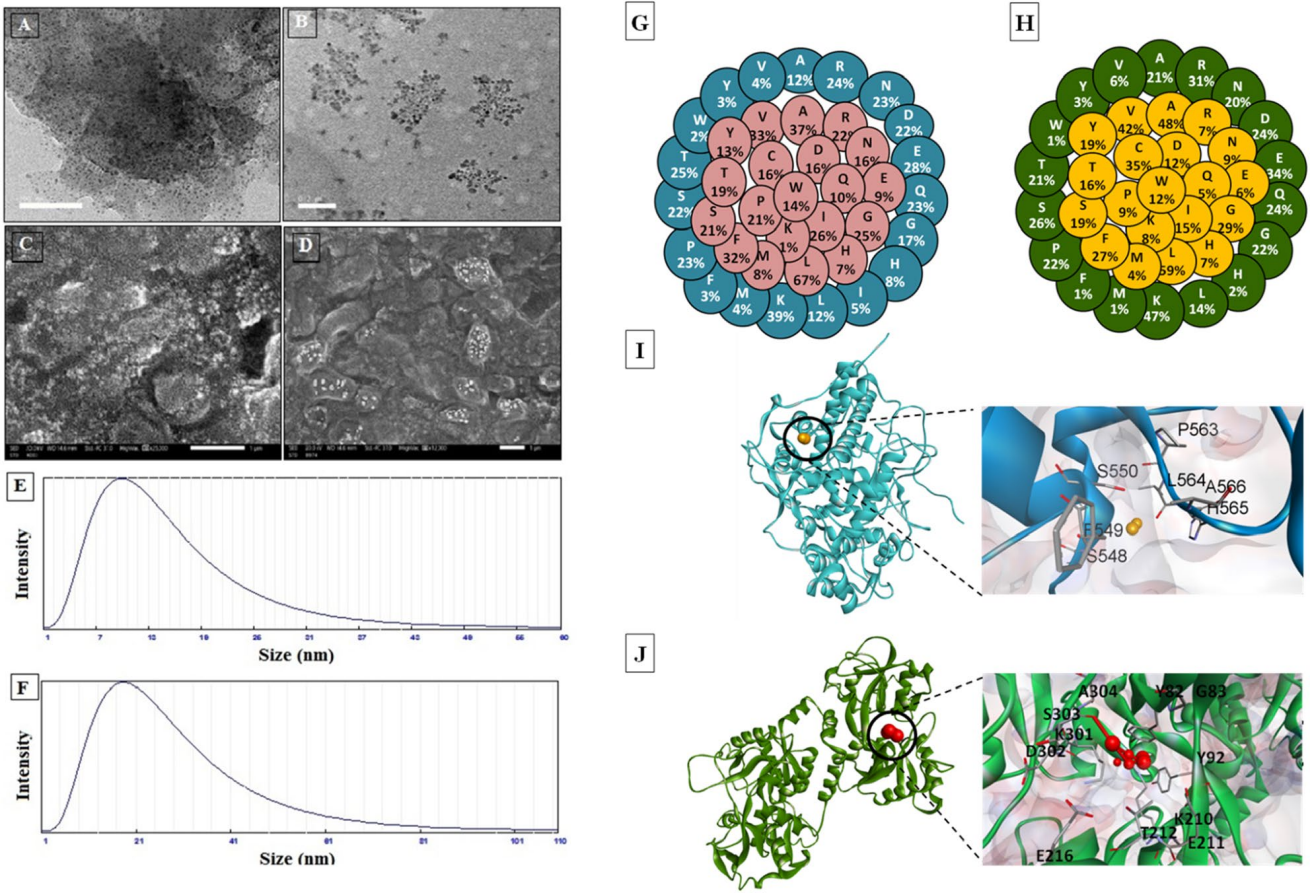
Characterization of nanofabricated LP and LF with hybrid Fe and Cu-NPs. (**A**,**B**) SEM micrographs of the nanofabricated LP with CNPs (LP-CNPs) and LF with FNPs (LF-FNPs), respectively. (**C**,**D**) TEM micrographs of the prepared LP-CNPs and LF-FNPs, respectively. (**E**,**F**) particle size distribution at angle 11.1° of the prepared LP-CNPs- and LF-FNPs, respectively. (**G**,**H**) Predicted exposed and buried amino acid residues in LP (Accession: AAA62714, 712 amino acids) and LF (Accession: AAA30610, 708 amino acids) via the DeepREx-WS webserver (https://deeprex.biocomp.unibo.it). (**I**,**J**) The LP-CNPs (LP "blue cartoon" with CuO "orange space-filling spheres style") and LF-FNPs (LF "green cartoon" and Fe_3_O_4_ "red space-filling spheres style") docked complex structures, respectively, as provided by the HDOCK server (http://hdock.phys.hust.edu.cn/) and were visualized by Discovery Studio 2020 Client software 20.1 (https://www.3ds.com/products-services/biovia/products/molecular-modeling-simulation/biovia-discovery-studio). The magnified regions are referred to as the interacting residues with CuO or Fe_3_O_4_ in the LP-CNPs and LF-FNPs docked complexes, respectively.

Figure 1A–D reveals the morphological characterization of both nanocombinations of LP with CNPs (LP-CNPs) and LF with FNPs (LF-FNPs). The scanning electron microscope (SEM) and transmission electron microscopy (TEM) micrographs indicated that the formed nanocombinations of both LP and LF are homogenous in particle size and uniformly distributed in LP-CNPs and LF-FNPs. As shown, the tiny spherical formulations of LP-CNPs- and LF-FNPs with particle sizes of 11.0 nm and 20.65 nm, respectively (Fig. 1E,F) were produced. In concern, Eltarahony et al.^41^ revealed that the synthesized FNPs using *S. cyaneofuscatus* EM3 were formed in tiny, numerous, roughly globular or quasi-spherical NPs in the range of 2–7 nm with a minor tendency for aggregation. However, they revealed that the synthesized hybrid CNPs showed irregular, undefined shapes and assembled in nanoclusters or bulks with an obvious appearance as opaque spots^41^. Both LP and LF were found to be coated on the surface of CNPs and FNPs to form the modified nanoformulating LP and LF (LP-CNPs and LF-FNPs, respectively). The recovered LP-CNPs and LF-FNPs were formed in a well-dispersed pattern and adhered-to protein moieties after nanofabrication (Fig. 1A–D). However, several previous studies reported the nanoformulating of LF using liposome, alginate-chitosan and iron oxide NPs^42–44^ and for nanoformulating LP using graphene oxide NPs, copper phosphate hybrid nanoflower, silica NPs and silver NPs^45–47^. There is only one study that nanoformulated LF with LP using chitosan, producing combined LP-LF NPs with size ≥ 460 nm^40^. These previously prepared combined NPs had > 23-folds-larger size than the currently prepared NPs. Besides the small sizes of these novel combined NPs that improve their cancer cell penetration, LP and LF were nanocombined with hybrid CNPs and FNPs, which exhibited unique and potent synergistic anticancer activity against human cancer cells, as evidenced recently^41^. Accordingly, these novel NPs could present powerful apoptotic agents as described below.

The computational analysis was used to predict the exposed and buried amino acid residues in the Bovine LP and LF proteins from their NCBI (the National Center for Biotechnology Information) database extracted sequences by the DeepREx-WS server. The outcomes found that there are 299 (41.99%) exposed and 413 (58.01%) buried residues in the LP, while LF contains 320 (45.20%)/388 (54.80%) exposed/buried residues. Results detected more than 72% of the polar amino acid residues on the surface of both proteins such as Asn, Asp, Lys, Glu, Gln, Arg, Thr, Ser, His, Tyr, and Met (Fig. 1G,H). The docking results of CuO with LP and Fe_3_O_4_ with LF revealed the presence of seven interacted amino acid residues with CuO at the LP interface and eleven interacted residues in the LF interface with the Fe_3_O_4_ (Fig. 1I,J). In this novel in vitro study, the most active anticancer milk proteins (LP and LF) were nanoformulated using CNPs and FNPs. Our results revealed that the interaction or adsorption of LP or LF on the surface of Cu or Fe-NPs enhances the formation of the modified LP-CNPs and LF-FNPs complexes which increases the stability and retention of both proteins, hinting at their uses in medicinal fields as potent anticancer candidates for the treatment of several cancer types. To explain the ability of LP and LF to interact with these metals, the current study predicted the type and content of the amino acids exposed on the surface of these proteins. Metal ions can bind directly to the amino acid functional groups, especially the carboxylate group of Asp and Glu, the imidazole group of His, and the aromatic side chain of Trp, Phe, and Tyr^48,49^. Other amino acids such as Asn, Gln, Lys, and Arg can be attributed indirectly to metal-chelating via single or compound impact on the His accessibility^49^. As shown in Fig. 1G,H, the exposed residues on the surface of both LP and LF include these metal-chelating amino acids. The previous studies reported the principal target of the carboxyl group in Asp and Glu to chelate Fe^3+^, while the imidazole group of His targets Cu^2+^ ions^48,49^. The interacting pocket in the LP-CNPs and LF-FNPs verified this, which comprised mainly Tyr 82, Tyr 92, Glu 211, Glu 216, Asp 302, Lys 210, Lys 301 in LF-FNPs, and Phe 549 and His 565 in LP-CNPs.

It was observed that there is no significant difference in the activities of free milk proteins and their nanoformulated with CNPs or FNPs for catalysing reduction and oxidation reactions (Table 1). Generally, both enzyme activity and stability are affected through nanofabrication and immobilization processes dependent on the used methods such as adsorption and/or entrapment. Based on this fact, in the present study, unlike the entrapment of LP and LF, the adsorption of LP and LF on the surface of NPs did not improve their activity may be owing to modifications in their physical and chemical properties as well as changes in their surface microenvironments. All of these may maintain the tertiary structure of active sites and substrate binding and affect their kinetic parameters. Meanwhile, the interaction of enzymatic residues and reactive groups of the adsorbed LP and LF on the NPs' surface may happen away from the active sites and substrate binding without altering their surface microenvironment^50^. Therefore, both LP-CNPs and LF-FNPs were able to maintain the activity of LP and LF during the nanofabrication process. However, many studies revealed that the activity of many proteins and enzymes has been demonstrated greater activity than their free form during formulation to chitosan-based NPs^51–53^.

**Table 1:** Activity of LP and LF (U/mg) in the presence of CNPs and FNPs.

Compound	LP	LP-CNPs	LF	LF-FNPs
Protein activity	6.74 ± 0.051	7.33 ± 0.096	10.69 ± 0.700	11.12 ± 0.117

All values are expressed as mean ± S.E.

CNPs: CuO nanoparticles; FNPs: iron nanoparticles; LF: lactoferrin; LP: lactoperoxidase.

### Growth inhibitory potential of LP-CNPs and LF-FNPs nanocombinations on cancer cells

**Figure 2:**
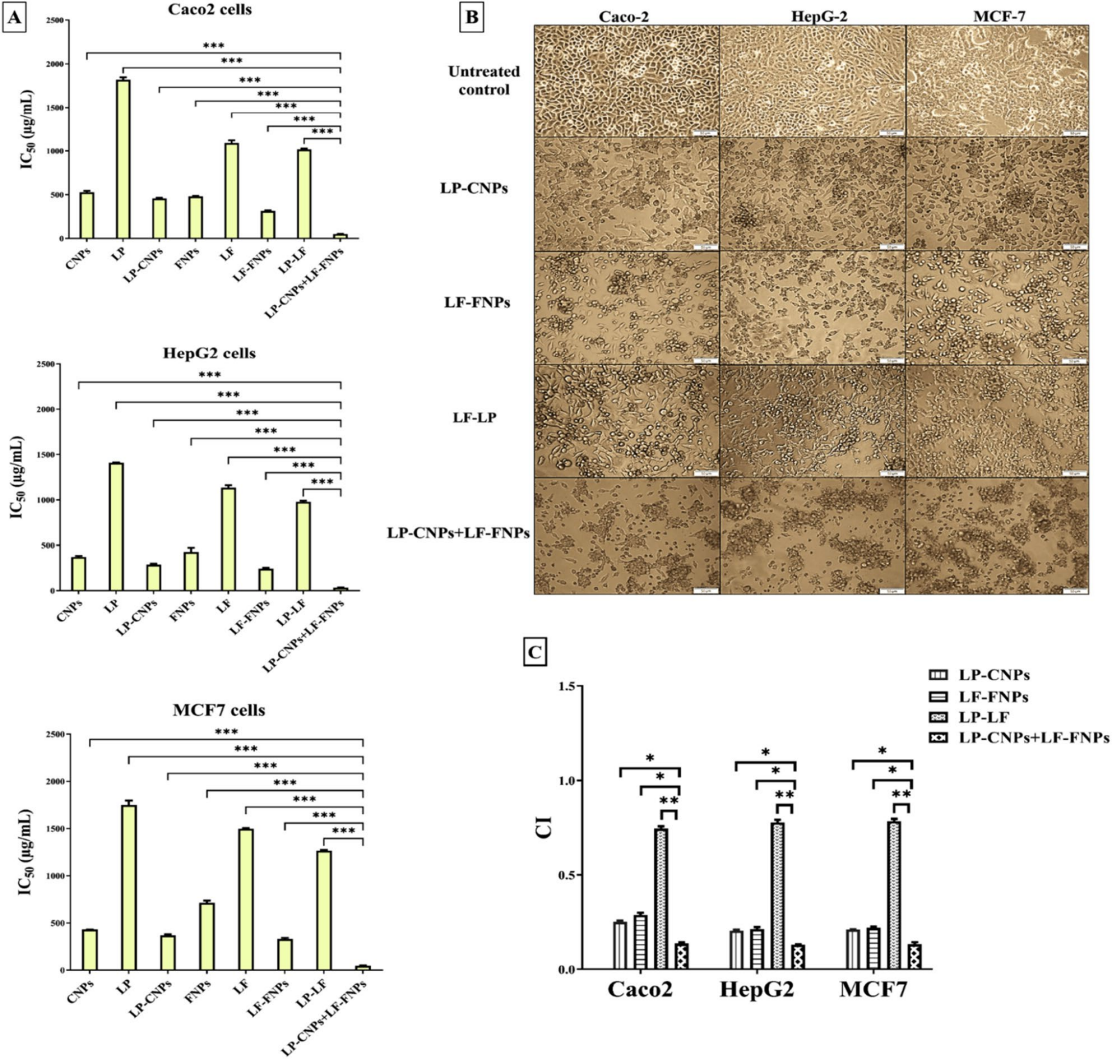
Cytotoxicity of LP and LF against human Caco2, HepG2 and MCF7 cancer cells in the presence of CNPs and FNPs. (**A**) IC_50_ of all prepared formulas for inhibiting growth of Caco2, HepG2 and MCF7 with (**B**) Morphological changes of human cancer cells after 72 h treatment with LP-CNPs, LF-FNPs, LF-LP and LP-CNPs + LF-FNPs (Magnification 100 X; scale bar 50 µm). (**C**) Combination index (CI) values of LP-CNPs, LF-FNPs, LF-LP and LP-CNPs + LF-FNPs. All values are demonstrated as mean ± S.E. LP-CNPs + LF-FNPs were compared to other formulas and considered statistically significant at p < 0.05*, p < 0.005**, p < 0.0005***. CNPs: CuO nanoparticles; FNPs: iron nanoparticles; LF: lactoferrin; LP: lactoperoxidase.

The impact of our novel nanocombinations on mediating selective apoptotic efficacy and impairing the migration potency of cancer cells was investigated. In the present study, all prepared formulas exhibited a growth inhibitory effect on Caco2, HepG2 and MCF7 in a dose-dependent manner (see Supplementary Fig. S1). Importantly, coating or adsorption of LP or LF on Cu and Fe nanometals was found to be strongly boosted their antitumor effects to be markedly more selective against cancer cells with high synergism as compared to free forms or combined proteins and other nanometals. Before comparing the anticancer activity of LP-CNPs and LF-FNPs to free proteins, their cytotoxicity against normal cells (Wi-38, MNCs, and Vero) was investigated. Among all prepared formulas, LP-CNPs + LF-FNPs and LP + LF had significantly the highest IC_50_-N values for all studied normal cells (Table 2). Notably, the binding of CNPs to LP and FNPs to LF improved their anticancer activity as evidenced by lowering IC_50_ values for LP-CNPs (< 460 μg/ml) and LF-FNPs (< 330 μg/ml) compared to LP (> 1408 μg/ml), LF (> 1090 μg/ml) and LP + LF (> 978 μg/ml). More importantly, LP-CNPs + LF-FNPs exhibited significantly the lowest IC_50_ values of 50.75, 34.09 and 46.04 μg/ml against Caco2, HepG2 and MCF7, respectively (Fig. 2A). Consequently, morphological alterations of the treated human cancer cells were more severe in the combined nanoformula of LP-CNPs + LF-FNPs than in the individual nanoformulas of LP-CNPs and LF-FNPs (Fig. 2B). The estimated CI values of all prepared nanoformulas were ≤ 0.3 reflecting a higher synergistic anticancer effect than LP + LF (CI ~ 0.8). LP-CNPs + LF-FNPs showed the maximum synergistic anticancer effect with the lowest CI values of 0.137, 0.129 and 0.133 against Caco2, HepG2 and MCF7, respectively (Fig. 2C). Moreover, these combined nanoformulations (LP-CNPs + LF-FNPs) demonstrated the highest SI, against cancer cells, compared to other formulas by more than fivefolds (Table 2). Both Cu and Fe are well-known as functional components in several cellular and metabolic processes, however, in high concentrations, they can undesirably affect^54^. In addition, due to the high stability of nanometals toward harsh circumstances such as pH intensifies and temperature increases their successful uses in many medical applications^55^. Herein, the synergistic effect of the nanoformulated LP and LF is may be related to the stability of the tested proteins after adsorption on the surface of NPs, besides it may be attributed to the change that occurred to the total bio-reactivity of the new formed NPs. Furthermore, the cationicity features of the proteins that coated Cu and Fe NPs make them able to disrupt the cell membrane of the treated cancer cells with high selectivity, sparing normal cells. This selectivity toward cancer cells due to the negative charge of the cell membrane in cancer cells is more than in normal cells, which increases their binding to the cationic peptides and proteins, consequently leading to death of the treated cancer cells without adverse action against surrounding normal tissues and cells^56^. Previous studies found that CuNPs synthesized by *Bacillus cereus* display a similar effect at IC_50_ ≥ 20 μg/mL on MCF-7, Caco-2 and HepG2 cells^52^, while CuNPs produced by *Sargassum polycystum* brown seaweed at IC_50_ = 61.25 μg/mL were effective on MCF-7^53^. On the other hand, Namvar et al.^54^ recorded the effect of IC_50_ of 18.75 μg/mL and 23.83 μg/mL of iron oxide (IO) NPs synthesized by *Sargassum muticum* (seaweed) on MCF-7 and HepG2 cells, respectively.

**Table 2:** IC_50_-N (μg/ml) and stimulation index (SI) of the prepared formulas.

Formulations	IC_50_-N			SI		
	Wi-38 cells	HMNCs	Vero cells	Caco2	HepG2	MCF7
CNPs	1371.5 ± 8.5***	2156.6 ± 11***	1291.5 ± 3.1***	2.601 ± 0.072***	3.693 ± 0.095***	3.185 ± 0.022***
LP	3082.5 ± 10.5**	3532.9 ± 20***	3156.9 ± 7.1***	1.696 ± 0.026***	2.188 ± 0.003***	1.762 ± 0.039***
LP-CNPs	3784 ± 14**	3964.1 ± 7.5***	3349.6 ± 15.2***	8.300 ± 0.071***	13.150 ± 0.238***	10.230 ± 0.273***
FNPs	2459.5 ± 9.5**	2484.5 ± 6.5***	2237.7 ± 7.9***	5.119 ± 0.059***	5.810 ± 0.487***	3.449 ± 0.093***
LF	4129 ± 9**	4383.7 ± 11**	4222.1 ± 4.3**	3.789 ± 0.082***	3.638 ± 0.065***	2.756 ± 0.004***
LF-FNPs	4134 ± 6**	4360.5 ± 6.3**	4217.2 ± 5.4**	13.199 ± 0.272***	17.162 ± 0.622***	12.576 ± 0.368***
LP-LF	5056 ± 33	5246.1.1 ± 9.4	5123.7 ± 1.3	4.982 ± 0.075***	5.173 ± 0.079***	3.998 ± 0.006***
LP-CNPs + LF-FNPs	5067.5 ± 55.5	5223.2 ± 4.6	5083.8 ± 13	66.725 ± 2.895	99.122 ± 1.046	73.604 ± 4.534

All values are expressed as mean ± S.E. LP-CNPs + LF-FNPs were compared to other formulas and considered statistically significant at p < 0.05*, p < 0.005**, p < 0.0005***.

CNPs: CuO nanoparticles; FNPs: iron nanoparticles; LF: lactoferrin; LP: lactoperoxidase.

### Proapoptotic potential of LP-CNPs and LF-FNPs nanocombinations with cell cycle arrest in the treated cancer cells

**Figure 3:**
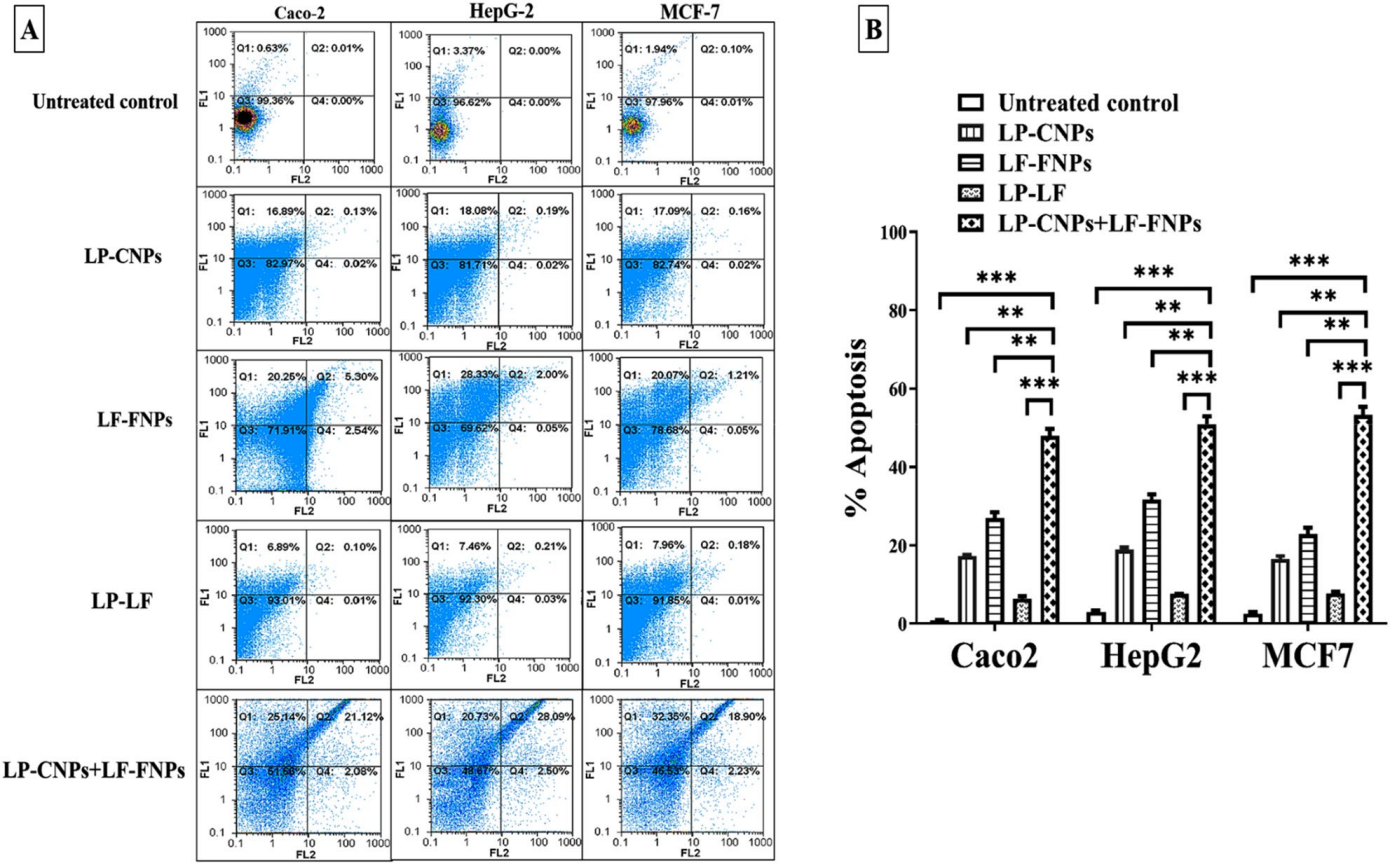
Flow cytometric analysis of apoptotic effect of the most effective anticancer nanoformulas and LP + LF. (**A**) Flow charts of LP-CNPs, LF-FNPs, LF-LP and LP-FNPs + LF-FNPs-treated Caco2, HepG2 and MCF7 cells relative to the untreated cells with (**B**) the percentage of total apoptotic cell population [% of both early and late apoptosis stages (annexin V-stained population “Q1” and annexin V/PI-stained population “Q2”, respectively)]. All values are demonstrated as mean ± S.E. LP-CNPs + LF-FNPs were compared to other formulas and considered statistically significant at p < 0.05*, p < 0.005**, p < 0.0005***. CNPs: CuO nanoparticles; FNPs: iron nanoparticles; LF: lactoferrin; LP: lactoperoxidase.

**Figure 4:**
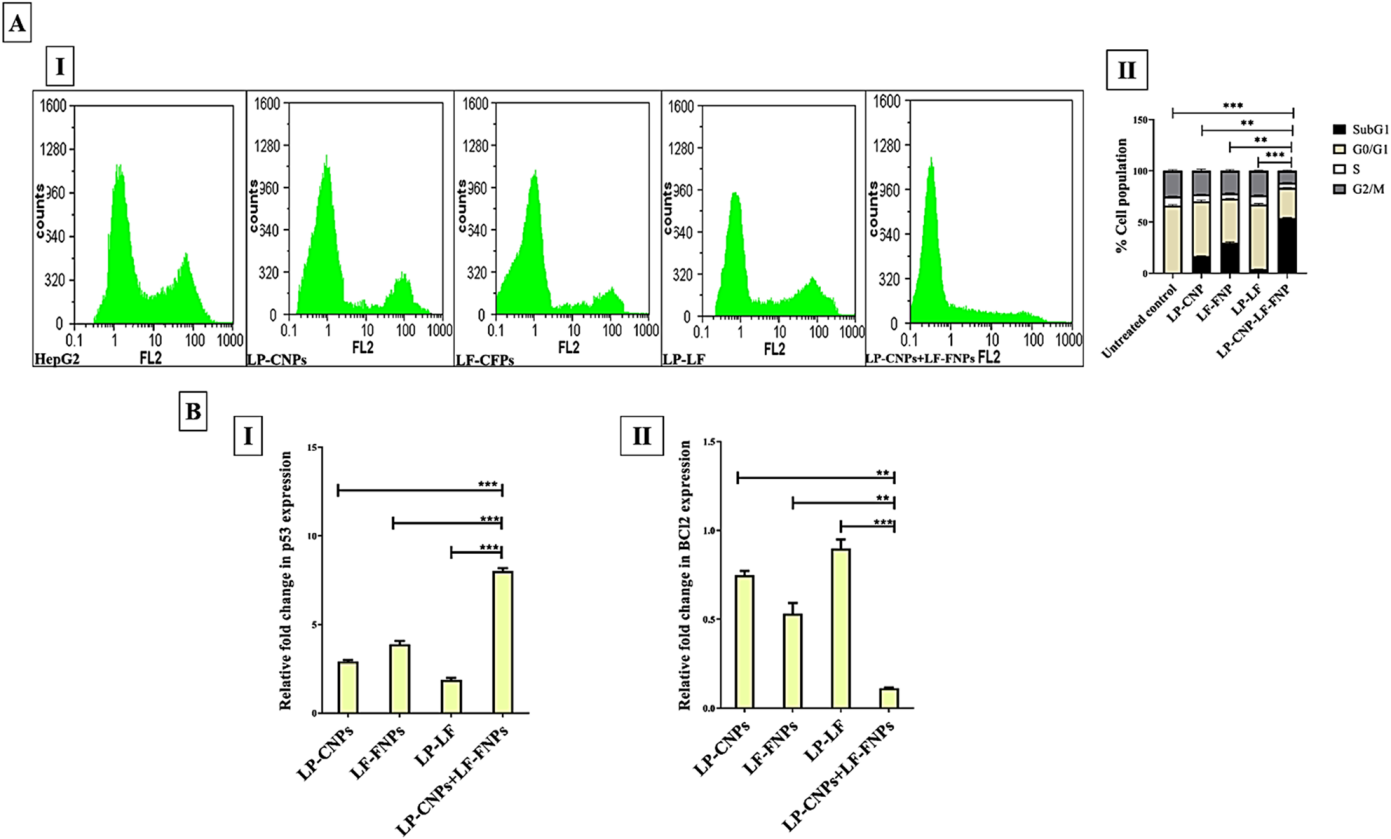
Alteration in cell cycle phases and in gene expression after treatment with the most effective anticancer nanoformulas and LP + LF. (**A**) Flow cytometry of cell cycle changes in treated HepG2 cells; (**I**) cell cycle charts with **(II)** percentages of cell populations in G0, G1, S, and G2/M. All values are demonstrated as mean ± S.E. LP-CNPs + LF-FNPs were compared to other formulas at G0 phase and statistically significant at p < 0.05*, p < 0.005**, p < 0.0005***. (**B**) Relative fold change in expression of (**I**) p53 and (**II**) BCl2 in MCF7 cells treated with LP-CNPs, LF-FNPs, LF-LP or LP-CNPs + LF-FNPs. All values are demonstrated as mean ± S.E. LP-CNPs + LF-FNPs are statistically significant with other formulas at p < 0.05*, p < 0.005**, p < 0.0005***. CNPs: CuO nanoparticles; FNPs: iron nanoparticles; LF: lactoferrin; LP: lactoperoxidase.

The apoptosis-mediated anticancer effect of the most active formulas (LP-CNPs, LF-FNPs, and LP-CNPs + LF-FNPs compared to LP + LF) was evaluated using flow cytometry of annexin-stained apoptotic cells and cell cycle alterations, qPCR, and immunohistochemical analyses. Flow cytometry was used to assess the percentage of the apoptotic population in the treated human cancer cell lines after staining with a dual nuclear dye of annexin V/PI. Apoptotic cells have a high affinity for binding with annexin V and these cells were stained with both annexin and PI at the late stage of apoptosis. Figure 3A,B illustrates that LP-CNPs + LF-FNPs had the highest potential to induce apoptosis in three cancer cell lines (47.98 ± 1.725, 50.86 ± 2.04 and 53.27 ± 2.02, respectively) compared to ≤ 19%, ≤ 32% and ≤ 8% for LP-CNPs, LF-FNPs and LP + LF, respectively. Importantly, cell cycle analysis illustrated that LP-CNPs + LF-FNPs-treated HepG2 cells had the highest percentage of G0 population (53.76 ± 0.535%) and the lowest percentage of G1 population (29.49 ± 0.405%) by more than twofolds compared to LP-CNPs, LF-FNPs, and LP + LF. Furthermore, HepG2 cells treated with LP-CNPs + LF-FNPs had the lowest percentages of S (5%) and G2/M (12%) populations, indicating cell cycle arrest (Fig. 4AI,II). These flow cytometric results were supported by the detection of relative change in the expression levels of two key genes involved in apoptosis and oncogenesis (p53 and BCl2, respectively). Figure 4BI illustrates that the p53 expression level was up-regulated by eightfolds in LP-CNPs + LF-FNPs-treated MCF7 cells compared to ~ 3-, 4- and 2-folds in LP-CNPs, LF-FNPs and LP + LF, respectively (see Supplementary Table S1). In terms of relative expression of BCl2, LP-CNPs + LF-FNPs exhibited the strongest potential to down-regulate it by tenfolds as it was shown in Fig. 4BII. Meanwhile, LP-CNPs, LF-FNPs and LP + LF suppressed BCl2 by lesser potency (≤ twofolds) (see Supplementary Table S1).

**Figure 5:**
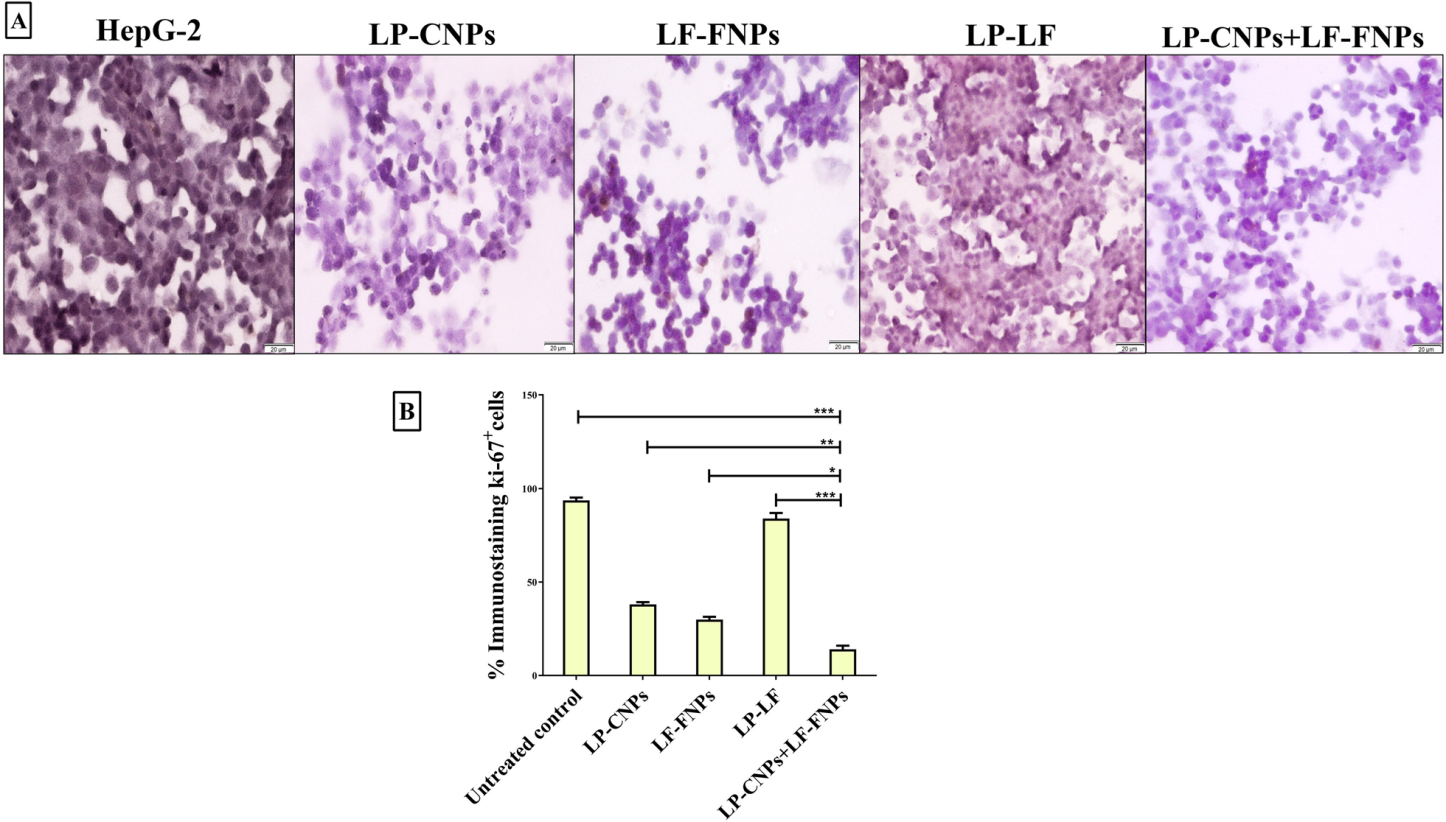
Immunohistochemical analysis of proliferation marker (Ki-67) in HepG2 cells after treatment with the most effective anticancer nanoformulas and LP + LF. (**A**) Immunohistochemical staining images of the untreated and LP-CNPs, LF-FNPs, LF-LP and LP-CNPs + LF-FNPs -treated HepG2 with (**B**) Ppercentage of Ki-67^+^-immunostained cell population in the untreated and treated HepG2 cells (Magnification 200 X; scale bar 20 µm). All values are demonstrated as mean ± S.E. LP-CNPs + LF-FNPs are statistically significant with other formulas at p < 0.05*, p < 0.005**, p < 0.0005***. CNPs: CuO nanoparticles; FNPs: iron nanoparticles; LF: lactoferrin; LP: lactoperoxidase.

Regarding the immunohistochemical analysis of proliferation marker (Ki-67), it was suppressed in all treated HepG2 cells, as illustrated by a decreased brown-colored product of secondary antibody relative to the untreated cells (Fig. 5A). LP-CNPs + LF-FNPs -treated HepG2 cells had the lowest percentage of the ki-67^+^-immunostained population (13.26 ± 1.37%) compared to 37.93%, 29.90% and 84.69% in LP-CNPs, LF-FNPs and LP + LF-treated cells, respectively (Fig. 5B).

Our results are in agreement with many previous studies that illustrated that LF and its derived peptides and fragments such as lactoferricin B stimulated apoptosis-mediated anticancer activity by inhibiting autophagy against many cancer types such as human gastric cancer^57^, colorectal cancer cells^58,59^, breast cancer^60^ and leukemia^61^. Other interesting results in a previous study demonstrated that LP exerts anti-tumor effect through its contribution to the apoptosis-dependent antineoplastic activity of iodide or iodine in an induced mammary cancer model in rats^62^. Moreover, previous findings demonstrated the synergistic p53-mediated apoptotic activity of chitosan nanocombination of LP and LF against Caco-2, HepG2, MCF-7, and PC-3 cell lines^40^. This previous study also illustrated that nanocombination of LP and LF-loaded chitosan induced cell cycle arrest in four above-mentioned cancer cells^40^. Eltarahony et al.^41^ found that hybrid CNPs-FNPs mediated caspase 3-dependent apoptosis in the treated human cancer cell lines. In the current study, the apoptotic mediated mechanism of both tested NPs and their combinations were observed through reduction of proliferation index (ki-67) and consequently upregulation of p53 and suppression of B-cell lymphoma 2 (Bcl-2) in all treated cancer cells. In agreement with our results, many studies have revealed that bovine LF exerts its anticancer activity against human breast and colon cancer cells by many diverse signaling mechanisms such as p53, Bcl-2, survivin and caspase-dependent apoptosis^58,60^. In addition, LP was found to be able to mediate its anticancer activity against the bone cell model through downregulation of nuclear factor (NF)-κB^63^. Our results reflected also the selectivity effect of the prepared nanocombinations of LP and LF with Cu and Fe nanometals toward cancer cells, which has been reported for metal oxides in recent studies^64,65^.

In previous studies, CuO nanometals have exerted their anticancer effect on myelogenous leukemia (K562) cells via suppression of mitochondrial pathways and down-regulation of tumor genes^66,67^. Significantly, p53 is one of the most oxidative stress response transcription parameters^68^. The up-regulation of the p53 gene and induction of the BAX ” Bcl-2 associated X protein”/Bcl-2 ratio assist to trigger the release of cytochrome c from the mitochondria, and enhancing the apoptotic pathway and caspase cascade^69^. In the other study, Azizi et al. revealed the prepared albumin-coated CuNPs exerted their anticancer activity through the apoptosis route, leading to cell cycle arrest in the treated MDA-MB-231 cell line^70^. This apoptotic effect was found to be based on induction of oxidative stress which was evident by reactive oxygen species generation. The use of LF nanoparticles has attracted much attention as carriers for targeted delivery of chemotherapeutic drugs, owing to the fact that they increase endocytic internalization of the drugs by brain cancer cells greater than surrounding normal cells^71,72^. Kanwar et al. demonstrated that the oral administration of Fe-bLf acts as a fortifying candidate for enhancing cancer treatment in mice model and increased the production of nitric oxide and many cytokines, including tumor necrosis factor, interferon-γ, which enhances the sensitivity of resistant tumours to widely used chemotherapeutics such as Doxorubicin^73^. Furthermore, both Apo-bLf and Fe-bLf were found to be rapidly internalised into osteoarthritis and colon cells, which are mediated by LF and transferrin receptors^74,75^. In this context, the ability of LF and LP to uptake rapidly by cancer cells due to the enhanced permeation and retention effect of LF nanocombinations mediated by the passive permeability of LF in the cancer cells^76^ like other proteins such as albumin^70,77^. Interestingly, LF and LP nanocombinations induce active absorption of the used drug by the cancer cells through their receptors. In addition, according to the obtained size of both LP and LF nancombinations in this study (11 nm and 21 nm, respectively), passive accumulation in cancer cells due to the permeability and retention effect is considered to be particularly advantageous^78^. On the other hand, due to the presence of a higher O_2_ concentration in the cell membrane compared to the cell media, both CNPs and FNPs are oxidized via their pass through the cell membrane, producing copper and iron ions. Consequently, disruption of the cellular membrane might have happened via a metal release process into the cell, which leads to the accumulation of H_2_O_2_ at the cellular membrane^79^. It can be suggested that the ultimate fate of both Cu and Fe nanometals upon uptake by the tumor cells will be degrading and producing Cu^2+^ and Fe^2+^ions. So, after having their anticancer activity against the treated cancer cell lines or tissues and being released from cells as ions, they will not exert any cytotoxic effect, due to Cu^2+^ and Fe^2+^ions are not cytotoxic at concentrations lower than 500 μM^41,70^.

### The predicted inhibitory effect of LP-CNPs, LF-FNPs and their combination on the AMPK activity

**Figure 6:**
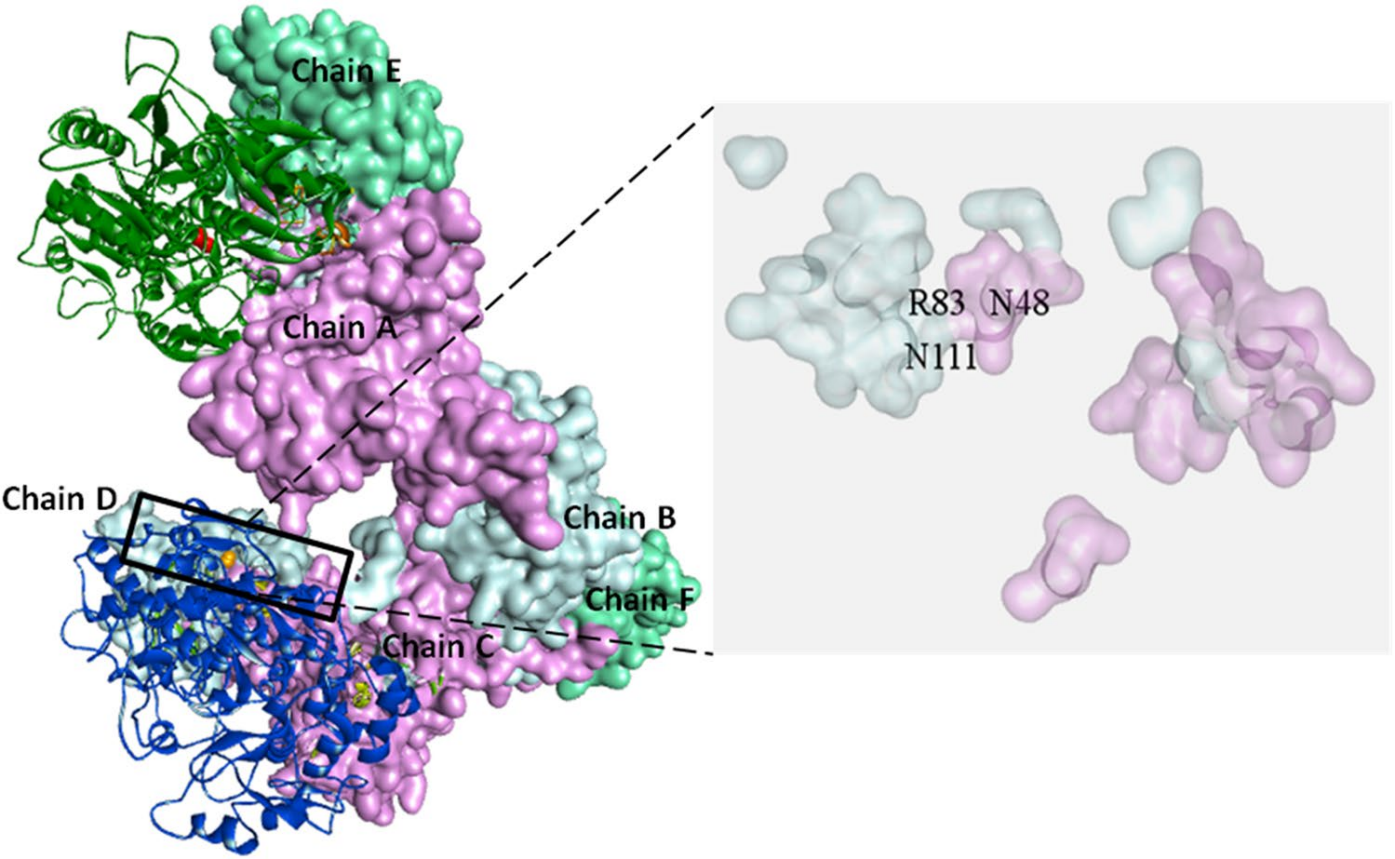
Docking model of AMP-activated protein kinase (AMPK, PDB ID: 4CFF, chain A, C "catalytic subunit, pink surface", chain B, D "beta1 subunit, light blue surface", chain E, F "gamma-1 subunit, light green surface") with LP-CNPs (blue cartoon, the orange space-filling spheres style refers to the interacting CuO) and LF-FNPs (green cartoon, the red space-filling spheres style refers to the interacting Fe_3_O_4_). The magnified residues elucidate the matched residues with the AMPK-activator (thienopyridone derivative) binding residues**.** The protein-metal docked complexes were given by the HDOOK server (http://hdock.phys.hust.edu.cn/), while protein–protein docked complexes were obtained by the PatchDock server (https://bioinfo3d.cs.tau.ac.il/PatchDock/php.php). All the provided docked complexes were visualized by by Discovery Studio 2020 Client software 20.1 (https://www.3ds.com/products-services/biovia/products/molecular-modeling-simulation/biovia-discovery-studio).

The proposed inhibitory impact of the LP-CNPs and LF-FNPs on the AMPK activity was performed using docking analysis. We choose this enzyme in our study due to its multi-anticancer effects on different types of malignancies^80^. The results revealed that LP-CNPs were able to interact with the catalytic subunit (chain A, C) and chain D of the beta-1 subunit of the enzyme. While LF-FNPs are bound to the chains C, D, as well as chain F of the gamma-1 subunit. In addition, docking outcomes reveal a higher binding affinity (ΔG) of LP-CNPs to AMPK (PDB: 4CFF) than that of LF-FNPs by 1.66-fold (Table 3). Therefore, docking of both the investigated metalloproteins to the AMPK began with LP-CNPs and then LF-FNPs to produce the AMPK_LP-CNPs_LF-FNPs docked complex. The binding of both metalloproteins to the AMPK exhibited a higher binding affinity than that of LP-CNPs or LF-FNPs alone by 1.69- and 2.82-folds, respectively (Table 3). The interacting pocket of the docked complexes was compared to the PDBSum-extracted active site residues and to the thienopyridine derivative (AMPK activator) binding site to distinguish the matched residues. The results revealed no matched active site residues, but three matched residues were detected between the interacting pocket of the docked complexes of AMPK_LP-CNPs and AMPK_LP-CNPs_LF-FNPs only and the enzyme activator binding site, as shown in Fig. 6.

**Table 3:** PDBePISA web server outcomes for the studied docked complex interfaces.

Docked complex	Δ^i^G, kcal/mol	iN_Res_	Interface area, Å^2^	N_HB_	N_SB_
AMPK_LP-CNPs	− 13.5	64	1927.2	22	1
AMPK_LF-FNPs	− 8.3	38	1345.1	7	4
AMPK_LP-CNPs _LF-FNPs	− 4.9	50	1667.3	17	0
Cathepsin B_LP-CNPs	− 19.7	76	2414.8	19	6
Cathepsin B_LF-FNPs	− 13.7	86	2689.2	14	6
Cathepsin B_LP-CNPs_LF-FNPs	− 27.5	115	3839.1	29	8
MMP-9_LP-CNPs	− 9.9	66	2348.2	22	14
MMP-9_LF-FNPs	− 20.2	79	2687.8	13	14
MMP-9_ LP-CNPs _LF-FNPs	− 23.2	145	4656	28	24

ΔG, binding affinity; ^i^N_res_, residues number; N_HB,_ potential hydrogen bond number; and N_SB,_ potential salt bridge number.

One of the important anticancer enzymes that can be activated by oxidative stress and other stress conditions is AMPK. This enzyme is a ser/thr protein kinase composed of a catalytic subunit (α) and two regulatory subunits (β and γ), and it is involved in a variety of regulatory processes that contribute to cancer progression. Hence, its activation causes virtually all cell growth anabolic pathways to be inhibited, directly inducing cell-cycle arrest by regulating p53 and other cell cycle co-regulators, and modulating inflammation^80^. Thus, AMPK activators could be a promising cancer treatment option. As a result of all of this enzyme's important anticancer effects, the current study used it for the molecular docking analysis to predict the influence of our studied metalloproteins on its activity. This will give more clarity to the apoptotic effect of these metalloproteins, besides the prediction of other anticancer mechanisms. The results of our study revealed that the combined form of these metalloproteins had the greatest binding affinity to AMPK and a proposed ability to boost its activity (Fig. 6, Table 3). These postulated outcomes give more importance to our investigated metalloproteins and their nanoformulations as promising anticancer agents.

### Anti-migration activity with the predicted inhibitory effect on the activity of the migration-related proteases

**Figure 7:**
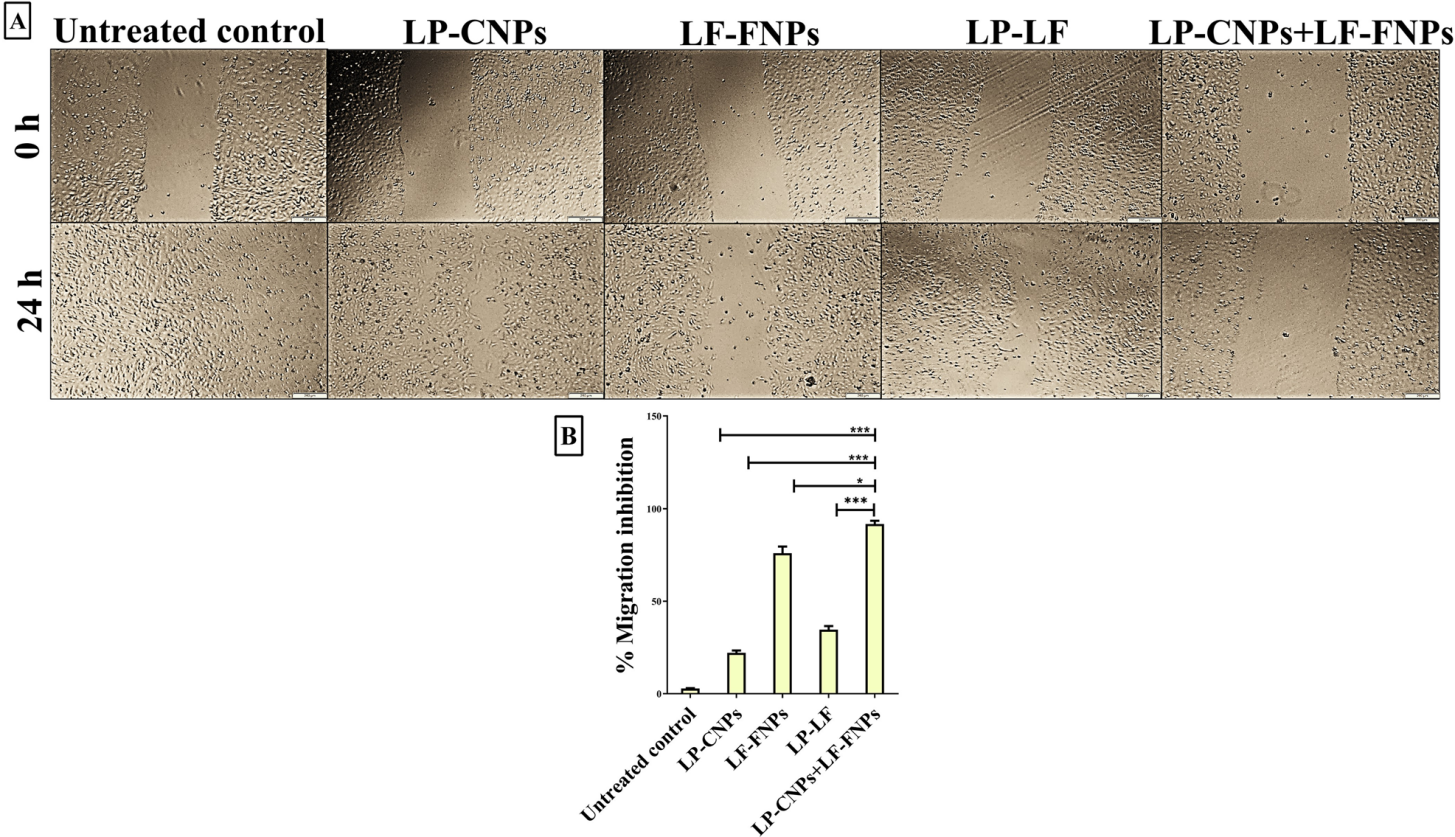
Anti-migration potency using wound healing assay. (**A**) Microscopic image of the scratched wound area in the untreated and LP-CNPs, LF-FNPs, LF-LP and LP-CNPs + LF-FNPs -treated Caco2 cells at 0 and 24 h (Magnification ×40; scale bar 200 µm); (**B**) relative migration inhibition percentages. All values are demonstrated as mean ± SE. LP-CNPs + LF-FNPs are statistically significant with other formulas at p < 0.05*, p < 0.005**, p < 0.0005***. CNPs: CuO nanoparticles; FNPs: iron nanoparticles; LF: lactoferrin; LP: lactoperoxidase.

Most importantly, the anti-metastatic activity of the above-mentioned formulas was determined using wound-healing assay. LP-CNPs + LF-FNPs exhibited the highest potency to inhibit migration of the treated Caco-2 by 91.80 ± 1.76%. Whereas LP-CNPs, LF-FNPs and LP + LF had lower efficacy to inhibit Caco-2 migration by 22.12%, 75.99% and 34.64%, respectively (Fig. 7A,B).

**Figure 8:**
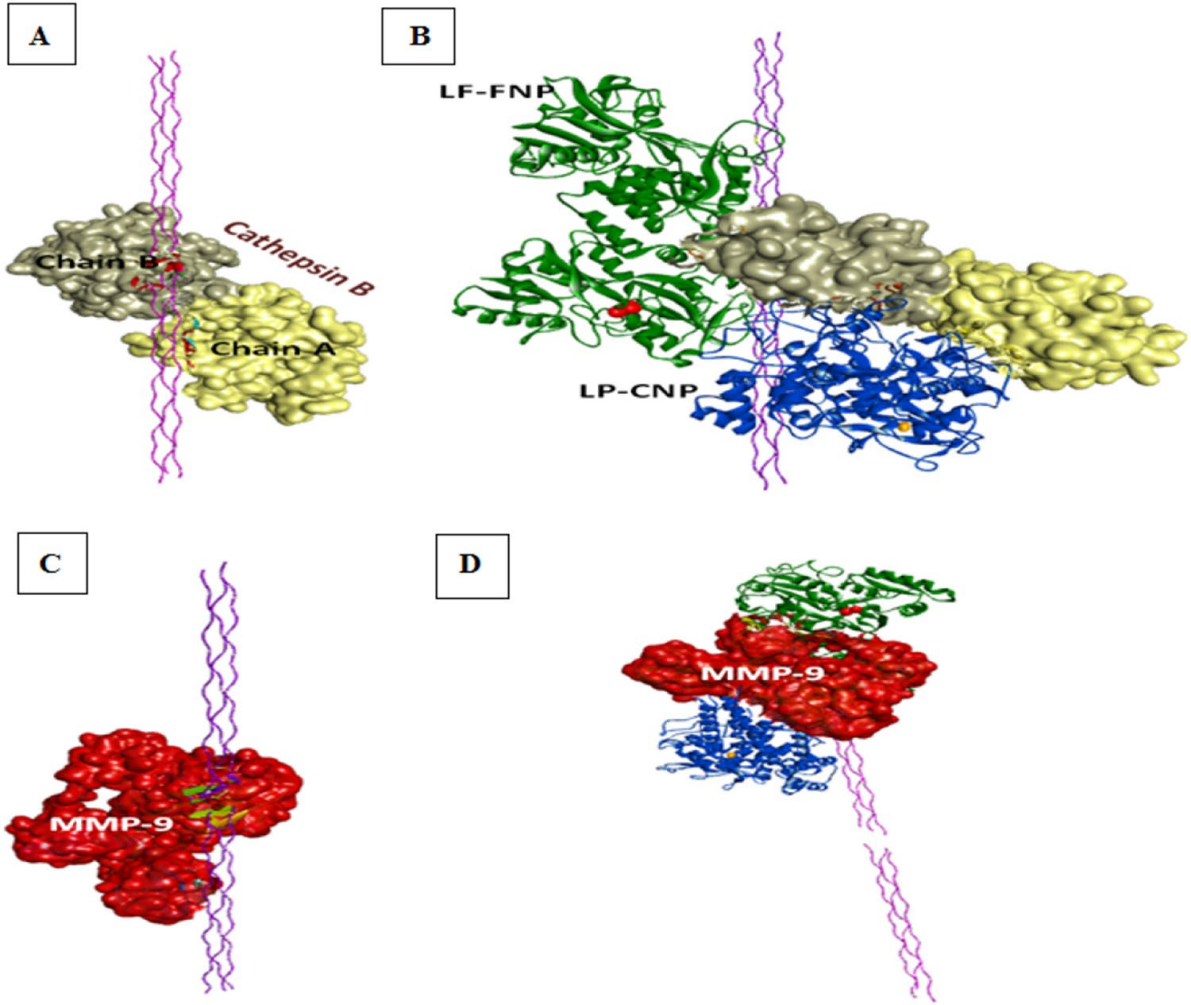
Proposed inhibitory effect of LP-CNPs and LF-FNPs combination on cathepsin B and matrix metalloproteinase (MMP)-9 activities. (**A**) Docking model of cathepsin B (PDB ID: 3AI8, chain A "yellow surface", chain B "gray surface") with collagen protein (triple helix model, PDB ID: 1K6F, purple). (**B**) Docking model of cathepsin B with LP-CNPs (blue cartoon, the orange space-filling spheres style refers to the interacting CuO) and LF-FNPs (green cartoon, the red space-filling sphere style refers to the interacting Fe_3_O_4_), followed by the collagen protein. (**C**) The docked complex of MMP-9 (PDB ID: 1L6J, red surface) with collagen protein. (**D**) Docking model of MMP-9 with LP-CNPs (blue cartoon, the orange space-filling spheres style refers to the interacting CuO) and LF-FNPs (green cartoon, the red space-filling sphere style refers to the interacting Fe_3_O_4_), followed by the collagen protein. The docked complexes of cathepsin B and MMP-9 with LP-CNPs and LF-FNPs were given by the PatchDock server (https://bioinfo3d.cs.tau.ac.il/PatchDock/php.php), while the docked complexes with collagen protein were obtained by the HDOOK server (http://hdock.phys.hust.edu.cn/). All the provided docked complexes were visualized by by Discovery Studio 2020 Client software 20.1 (https://www.3ds.com/products-services/biovia/products/molecular-modeling-simulation/biovia-discovery-studio).

The predicted suppressing capability of LP-CNPs, LF-FNPs, or the combination between them on the activity of the vital migration-related proteases (cathepsin B and MMP-9) was also evaluated here. The results elucidated the ability of the metalloproteins to bind to these important enzymes with different affinities. Hence, LP-CNPs showed a higher binding affinity to cathepsin B than that of LF-FNPs by 1.44-fold, while vice versa (2.04-fold) was noticed with MMP-9 (Fig. 8A–D, Table 3). Thus, docking of cathepsin B was started with LP-CNPs and then LF-FNPs, and the reverse was done for MMP-9. The binding of both metalloproteins to these proteases exhibited a higher binding affinity than that of LP-CNPs (1.4- and 2.34-folds, respectively) and LF-FNPs (2.01- and 1.15-folds, respectively) alone (Table 3). The current study compared the binding pockets in the obtained docked complexes with the PDBSum-provided active site residues of cathepsin B or MMP-9. The results showed the presence of matched residues with cathepsin B_LP-CNPs (H199A), cathepsin B_LF-FNPs (S25B, T120B), cathepsin B_LP-CNPs_LF-FNPs (H199A), and MMP-9_LF-FNPs _LP-CNPs (R134) docked complexes interface. However, neither MMP-9_LP-CNPs nor MMP-9_LF-FNPs docked complexes pocket residues revealed matched residues with the MMP-9 active site. Furthermore, the present study evaluated the influence of binding both LP-CNPs and LF-FNPs to cathepsin B or MMP-9 on the collagen (the main substrate) interaction (Fig. 8A–D). The results revealed the proposed efficiency of these metalloproteins (combined form) in decreasing the binding affinity between cathepsin B or MMP-9 and collagen protein by 3.24 and 17.50-folds, respectively (Table 3).

Furthermore, the present study utilized docking analysis to predict the impact of LP-CNPs and LF-FNPs on the activities of cathepsin B and MMP-9 for further explanation of their anti-migration influences against the different studied cell lines. These enzymes are activated in various types of cancer cells as a result of their acidic microenvironment and induce the destruction of the extracellular matrix, mainly the collagen protein, to promote metastasis^81,82^. Previous studies reported the link between high cathepsin B expression in hepatocellular carcinoma (HCC) patients and histological grade, advanced clinical staging, and tumor recurrence, suggesting that it could be a useful prognostic marker for these patients. Cathepsin B was also found to upregulate the expression of MMP-9 in HCC^83^. Furthermore, these enzymes were correlated with the poor prognosis of colon and breast cancer^84–86^. Our predicted outcomes revealed the highest binding affinity of the combined form of the examined metalloproteins to both cathepsin B and MMP-9 (Table 3) and showed a predicted inhibitory effect on these enzymes. Analysis of the interacting pocket in the obtained docked complexes showed the ability of the combined metalloproteins to bind with one active site residue in either cathepsin B or MMP-9, indicating the proposed competitive inhibitory action of these metalloproteins. Moreover, the binding of LP-CNPs and LF-FNPs to each of these enzymes substantially (3.24- and 17.50-folds, respectively) reduced their binding affinity to their main substrate, collagen (Table 3). As a result, the authors proposed that these metalloproteins might have another way of inhibition, such as a non-competitive mechanism. Thus, our nanoformulations incorporating these metalloproteins could be a novel therapeutic strategy for improving cancer prognosis.

## Conclusion

The results illustrated here propose exploiting both LP and LF nanocombinations with Cu and Fe (LP-CNPs and LF-FNPs) for their capability to provide a potent synergistic anticancer activity against different types of cancer cell lines with reduced adversity effects on the normal cells. The potent anticancer effects of LP and LF nancombinations could be attributed to the highest efficacy for inducing apoptosis with downregulating key oncogenes and enhancing proapoptotic genes in the treated human cancer cells compared to free or nanoforms of the studied proteins and nanometals. We strongly believe that the capability of LP and LF nanocombinations to target different types of cancer cells with a great degree of selectivity may provide dose reduction with increasing the lifetime survival of LP and LF. These results offer support for future anticancer uses of these nanocombinations using different animal models.

## Methods

### Purification of lactoperoxidase (LP) and apolactoferrin (LF)

Both bovine LP and LF were purified in our lab as previously described by Abu-Serie and El-Fakharany^40^. In brief, bovine skim milk was obtained by defatting and decaseination of raw milk via centrifugation at 5000 rpm for 30 min and decreasing the pH to 4.2 with 1 M HCl solution, respectively^87,88^. The supernatant of skim milk was dialyzed against 50 mM Tris HCl buffer, pH 8.0. The skim milk was applied into the pre-equilibrated Mono S 5/50 GL column with 50 mM Tris HCl buffer, pH 8.0. Both LP and LF were eluted with the same buffer containing 0.0–1.0 M NaCl. The pooled fractions of LP or LF were dialyzed in 50 mM phosphate buffer pH, 7.2 overnight and applied into a Sephacryl S100 column (5 × 150 mm, GE Health care, Sweden) equilibrated with 50 mM phosphate buffer pH, 7.2 containing 150 mM NaCl and eluted with the same buffer. The homogeneity and purity of both LP and LF were evaluated by 12% SDS-PAGE. All fractions of the purified LP and LF were pooled separately, dialyzed, lyophilized, and kept at − 80 °C for further uses.

### Biosynthesis of CNPs and FNPs

The biofabrication of both nanocomposites was performed in our lab according to the method followed by Eltarahony et al.^41^. In brief, *Streptomyces cyaneofuscatus* EM3 (accession number KY964508) was inoculated into 70 mL of starch-nitrate broth media containing 1 mM of each Fe(NO_3_)_3_·9H_2_O, Cu(NO_3_)_2_ and/or both in 250 mL Erlenmeyer flasks and incubated for 96 h at 30 °C with contentious shaking at 150 rpm. Media containing bacteria without metal precursors and media containing metal precursors without bacteria were included as the control samples. The synthesis of both NPs was detected through changes in biomass and media colours during the incubation period. To obtain bacteria biomass containing NPs, the bacterial cultures were harvested by centrifugation at 10,000×*g* for 20 min and washed three times with distilled water to remove media residues. Then, both CNPs and FNPs were extracted via homogenizing the cultural suspensions using lysis buffer and sonication at 60–80% amplitude and frequency of 20 kHz with 0.6 s pulse rate for 5 min. To obtain homogeneous mixtures, the lysed suspensions were exposed to a vigorous vortex for 5 min. At the end, the extracted NPs were obtained by centrifugation at 10,000×*g* for 20 min.

### Nanoformulation of LP using CNPs

This method was performed according to the method described previously^89^ with some modifications. LP (1 g/ml) was added dropwise to CNPs (1 mg/ml) in sodium phosphate buffer (pH 6.8) for 1 h with continuous stirring. The produced nanoformulated LP-CNPs were obtained after centrifugation and washing with phosphate buffer saline (PBS). Then it was freeze-dried using lyophilization before being stored in the refrigerator for further analyses. To confirm that all used amount of LP was nanoformulated with CNPs, the Bradford assay was used to detect protein concentration in supernatant^90^.

### Nanoformulation of LF using FNPs

LF (1 g/ml) was added dropwise to FNPs (1 mg/ml) in sodium phosphate buffer (pH 6.8) and was stirred for 1 h. After centrifugation, LF-FNPs were obtained and washed twice with PBS then freeze-dried. The Bradford assay was used to measure protein concentration in the supernatant to affirm that all of the LF was nanoformulated with FNPs^90^.

### Characterization of nanoformulated proteins (LP-CNPs and LF-FNPs)

The features of CNPs and FNPs were characterized previously as mentioned by Eltarahony et al.^41^. However, upon nanoformulation by LP and LF, their properties were characterized by SEM (JEOL JEM-1230, Japan), TEM (JEOL JEM-1230-Japan) and a particle size analyzer.

### Determination of LP and LF activity in the presence of CNPs and FNPs

Activity of LP and LP-CNPs was determined according to Chance and Maehly^91^. This assay is based on the activity of LP to oxidize guaiacol to tetraguaiacol using H_2_O_2_. LP or LP-CNPs were mixed with guaiacol (substrate) and H_2_O_2_ in 30 mM sodium phosphate buffer (PH 6) and the change in absorbance was measured at 470 nm for 3 min. One unit (U) of enzyme activity was calculated as the concentration of enzyme catalyzing the conversion of 1 μmol guaiacol to tetraguaiacol per min using the molar extinction coefficient of tetra-guaiacol of 26.6 mM^−1^ cm^−1^. Regarding the assessment of LF activity, LF or LF-FNPs were incubated with 300 µM nitroblue tetrazolium (NBT), NADPH and 30 µM of phenazine methosulfate (PMS) then absorbance was measured at 580 nm at 0 and 5 min. LF activity was calculated in U per milligram of protein using the standard curve of NBT^92^.

### Cell culture and media

The normal human lung fibroblast (Wi-38, CCL-75), African Green Monkey kidney Vero (CCL-81), colon cancer (Caco-2, HTB-37), liver cancer (HepG-2, HB-8065), and breast cancer (MCF7, HTB-22) cell lines were obtained from the American Type Culture Collection (ATCC, USA). Wi-38 cells (passage no. 34) and Caco-2 cells (passage no. 40) were cultured in Gibco Dulbecco's Modified Eagle Medium (DMEM, Lonza, USA)-contained 10% fetal bovine serum (FBS; Gibco, UK), 1% l-glutamine, and penicillin–streptomycin (100 U/ml of each). Vero cells (passage no. 39) were cultured in the same DMEM medium as previously mentioned, but with 5% FBS added. Meanwhile, HepG-2 (passage no. 51) and MCF7 (passage no. 43) cell lines were maintained and cultured in RPMI-1640 (Lonza, Switzerland) supplemented with 10% fetal bovine serum, 1% l-glutamine, and penicillin–streptomycin (100 U/ml of each). All used cell lines were seeded in polystyrene T-flasks, incubated in an incubator (5% CO_2_, 37 °C, and 95% humidity) and subcultured, using trypsin, at least twice before performing the following assays. Human blood mononuclear cells (HMNCs) were isolated from peripheral blood, which was collected from healthy volunteers. This is in accordance with the guidelines of the Research Ethical Committee, Faculty of Medicine, Alexandria University (Approval no. 0305246) and the recommendations of the National Health and Medical Research Council policies and the Ministry of Health and Population, Egypt.

### Cytotoxicity of the prepared formulations on normal human cell line

The cytotoxicity of CNPs, FNPs, LP, LP-CNPs, LF, LF-FNPs, LP + LF, and LP-CNPs + LF-FNPs were assessed using Wi-38, Vero, and HMNCs. The latter were isolated from heparinized blood samples. Briefly, these samples were carefully layered on Ficoll-Hypaque and centrifuged at 2000 rpm for 30 min. The undisturbed HMNCs layer was carefully collected and centrifuged twice at 1650 rpm for 5 min, then the cell pellet was suspended in RPMI-1640 medium containing 10% FBS and counted using the trypan blue exclusion method. Both Wi-38 and Vero cell lines were seeded, as 10^4^ cells/well, in polystyrene poly-d-Lysine-coated cell culture 96 well plates, while HMNCs were seeded, as 10^5^ cells/well, in 96 well U-bottom sterile cell culture plate. Five serial concentrations (2000, 1000, 500, 250, and 125 μg/ml) of the tested formulations were incubated with each of the studied normal cells for 72 h. Then cell viability was assayed by MTT (3-(4,5-dimethylthiazolyl-2)-2, 5-diphenyltetrazolium bromide) method^93^. Twenty microliters of 5 mg/ml MTT (Sigma, USA) were added to each well and the plate was incubated at 37 °C for 3 h. Then MTT solution was removed, 100 µl dimethylsulfoxide (DMSO) was added and the absorbance of each well was measured with a microplate reader (BMG LabTech, Germany) at 570 nm. The concentrations of the tested formulations at 50% cell viability (IC_50_-N) were determined by the Graphpad Instat software 3.0 (https://www.graphpad.com/scientific-software/instat/).

### Determination of the anticancer activity

#### MTT assay

Anticancer effect of the above-mentioned formulations was assayed using three human cancer cell lines (Caco-2, HepG-2, and MCF-7). All cancer cells (5 × 10^3^ cells/well) were seeded in sterile 96-well plates. After 24 h, serial concentrations of the tested formulations were incubated with three cancer cell lines for 72 h at 37 °C in a 5% CO_2_ incubator. MTT method was done as described above. Graphpad Prism software 9.0 (https://www.graphpad.com/scientific-software/prism/) was used to calculate the IC_50_ values that resulted in 50% of cancer death utilizing non-linear regression (dose–response curve was drawn as log inhibitor vs. normalized response with variable slope). Moreover, the selectivity index (SI) of all formulations was estimated as ratio between IC_50_-N of Wi-38 and IC_50_ of each cancer cell line as well as combination indexes (CI) of the combined formulations (LP-CNPs, LF, LF-FNPs, LP + LF and LP-CNPs + LF-FNPs) were calculated. These estimated CI values are indicators of the extent of the synergistic anticancer activity between metal oxide NPs and milk proteins. The CI value may be < 1 or > 1 or = 1 indicating a synergistic, antagonistic or additive effect, respectively. Additionally, the morphological alterations of the treated cancer cells relative to the untreated cancer cells were investigated using phase-contrast inverted microscope with a digital camera (Olympus, Japan).

#### Flow cytometric analysis of the apoptosis-mediated anticancer effect

The most effective formulations were selected to investigate their proapoptotic effect by incubation with three human cancer cell lines (Caco2, HepG2 and MCF7) for 72 h, at their IC_50_ doses (51, 34 and 46 μg/ml, respectively). Following the protocol of the Annexin-binding Buffer for flow cytometry kit (Invitrogen, UK), the untreated and treated cancer cells were suspended in a 100 μl binding buffer after trypsinization. Then 5 μl fluorescein isothiocyanate (FITC)-annexin V and 5 μl propidium iodide (PI, 2 μg/ml) was added and incubated for 15 min, in dark. Afterwards, cells were washed with PBS and suspended in a binding buffer. The annexin-stained apoptotic population was then quantified using flow cytometry at the FITC signal detector (FL1) against the phycoerythrin emission signal detector (FL2) for PI-stained necrotic cells and analyzed with FloMax software 2.3 (https://www.yumpu.com/en/flomax).

#### Flow cytometry analysis for cell cycle alteration

The change in the cell cycle distribution (G_0_, G_1_, S, and G_2_/M) in the untreated and treated HepG2 cells was investigated by incubating with 5 μg/mL RNase A and then 1 mg/mL of PI. Following washing, the cells were examined using flow cytometry at 488 nm with FloMax software 2.3 (https://www.yumpu.com/en/flomax).

#### qPCR analysis for the relative change in p53 and BCl2 expression

Total RNAs of untreated and the treated MCF-7 cells were extracted using the Gene JET RNA Purification Kit (Thermo Scientific, USA). The cDNA was synthesized from mRNA using the cDNA Synthesis Kit (Thermo Scientific, USA). Real-time PCR was performed using SYBR green master mix and specific primers (Forward/Reverse) for p53 and Bcl2 were 5′-ATGTTTTGCCAACTGGCCAAG-3′/5′-TGAGCAGCGCTCATGGTG-3′ and 5′-TCCGATCAGGAAGGCTAGAGTT-3′/5′-TCGGTCTCCTAA-AAGCAGGC-3′, respectively. The 2^−ΔΔCT^ equation was used to estimate the change in gene expressions in the treated cancer cells relative to untreated cancer cells.

#### Immunohistochemical analysis of proliferation index (ki-67)

After trypsinization, the untreated and treated HepG2 cells were centrifuged, washed and fixed with 10% formalin. The fixed cell specimens were dehydrated and immersed in xylene followed by impregnation in melted paraffin to form solid paraffin blocks. Then a rotator microtome was used to cut each block into 3–5 μm thick sections that were transferred into positively charged slides. Slides were dried, dewaxed, rehydrated, incubated in 3% H_2_O_2_ for 10 min, washed in PBS buffer twice for 3 min and put in 10 mM citrate buffer (pH) followed by heating for 10–20 min. After cooling and washing, slides were separately soaked overnight in primary antibody (anti-ki-67). Slides were washed, and covered with biotinylated goat anti-polyvalent secondary antibody for 10 min and then streptavidin peroxidase was added. After 10 min, the substrate of secondary antibody (3,3′-diaminobenzidine) was added to detect the percentage of ki-67^+^-immunostained cells by recording using imaging analysis cellSens software 1.16 (https://www.olympus-lifescience.com/en/software/cellsens) of phase-contrast microscope (Olympus, Japan).

#### Wound healing assay for detection of anti-migration inhibition potency

Caco-2 cells were seeded in 6-well plates and scratched with a sterile tip when they reached 90% of confluence. At 0 and 24 h, the wound closure area was measured and recorded by imaging analysis cellSens software 1.16 (https://www.olympus-lifescience.com/en/software/cellsens). The inhibition in area closure was calculated using ImageJ NIH software V1.61 (https://imagej.nih.gov/nih-image/index.html) to estimate the inhibition migration percentages in the treated wells versus untreated wells**.**

### In silico analyses

#### Prediction of exposed/buried residues in LP and LF proteins

The type and number of the buried and exposed residues in both LP and LF proteins were predicted via the DeepREx-WS web server (https://deeprex.biocomp.unibo.it)^94^ which allows for more accurate characterization of these regions. The Fast Adaptive Shrinkage Thresholding Algorithm (FASTA) format of LP (Accession: AAA62714, 712 amino acids) and LF (Accession: AAA30610, 708 amino acids) sequence was provided from the NCBI protein database (https://www.ncbi.nlm.nih.gov/protein/) and used for this analysis.

#### Protein modelling and compounds structures

The 3D structures of the bovine LF (PDB: 1BLF), bovine LP (PDB: 4GM7), human AMPK (PDB: 4CFF), human cathepsin B (PDB: 3AI8), human MMP-9 (PDB: 1L6J) and collagen triple helix model (PDB: 1K6F) were extracted from the Protein Data Bank (PDB, https://www.rcsb.org/). The 2D structures of CuO (CID_14829) and Fe_3_O_4_ (CID_16211978) were provided from the PubChem database (https://pubchem.ncbi.nlm.nih.gov/) and were included.

#### Molecular docking analyses

Docking of LP protein with CuO and LF with Fe_3_O_4_ was accomplished using the HDOOK server (http://hdock.phys.hust.edu.cn/) to produce LP-CNPs and LF-FNPs docked complexes, respectively. In addition, the molecular docking of LP-CNPs and LF-FNPs or both with each of the studied enzymes (AMPK, cathepsin B, and MMP-9) was performed separately using the PatchDock web server (https://bioinfo3d.cs.tau.ac.il/PatchDock/php.php)^95^ at clustering RMSD (root-mean-square deviation) default value (4). Then the top-scored docked complex was selected for further examination. The order of docking for both the studied metalloproteins to the investigated enzymes was related to their binding affinity to each of these enzymes. Also, cathepsin B and MMP-9 as well as their docked complexes with the LP-CNPs and LF-FNPs combined form were docked separately with the collagen protein using HDOOK server. The interfaces of the produced docked complexes were analyzed and visualized by the Discovery Studio 2020 Client program (v20.1.0.19295).

#### Binding affinity assessment in the docked complexes

The produced docked complexes were further analyzed using the PDBePISA (Proteins, Interfaces, Structures, and Assemblies) server (https://www.ebi.ac.uk/msd-srv/prot_int/cgi-bin/piserver)^96^. This web server provides the change in Gibbs free energy (ΔG, solvation-free energy) achieved upon the interface formation, as well as the number of potential hydrogen bonds, potential salt bridges, residues, and the area across the interface. The protein–protein binding affinity in the docked complex was deduced from the ΔG value.

#### Active site prediction

Using the protein PDB ID, the active site residues of AMPK, cathepsin B, and MMP-9 were supplied from the PDBsum web-based database (http://www.ebi.ac.uk/pdbsum)^97^ that contains the structural data on the PDB database entries.

### Statistical analysis

Data were expressed as mean ± standard error of the mean (S.E). Statistical significance was estimated by the multiple comparisons Tukey posthoc analysis of variance (ANOVA) using the SPSS16 program. The differences were considered statistically significant at p < 0.05.

## Supplementary Information

Supplementary Information.

## Data Availability

All data generated or analyzed during this study are included in this published article [and its supplementary information files].
