# Author Correction: Augmenting apoptosis-mediated anticancer activity of lactoperoxidase and lactoferrin by nanocombination with copper and iron hybrid nanometals

**DOI:** 10.1038/s41598-025-07601-6

**Published:** 2025-06-24

**Authors:** Esmail M. El-Fakharany, Marwa M. Abu-Serie, Noha H. Habashy, Marwa Eltarahony

**Affiliations:** 1 Protein Research Department, Genetic Engineering and Biotechnology Research Institute (GEBRI), City of Scientific Research and Technological Applications (SRTA-City), New Borg El-Arab, 21934 Alexandria Egypt; 2 Medical Biotechnology Department, Genetic Engineering and Biotechnology Research Institute (GE-BRI), City of Scientific Research and Technological Applications (SRTA-City), New Borg El-Arab, 21934 Alexandria Egypt; 3 Biochemistry Department, Faculty of Science, Alexandria University, New Borg El-Arab, 21511 Alexandria Egypt; 4 Environmental Biotechnology Department, Genetic Engineering and Biotechnology Research Institute (GEBRI), City of Scientific Research and Technological Applications (SRTA-City), New Borg El-ArabAlexandria, 21934 Alexandria Egypt

Correction to: *Scientific Reports* 10.1038/s41598-022-17357-y, published online 01 August 2022

The original version of this Article contained errors.

As a result of an error during figure assembly, Figure 5A used incorrect data from the Authors’ earlier publication^1^. This figure has now been updated with the correct data. Figure 5B was correct at the time of publication.

Additionally, in Figure 7A the data corresponding to 0h/LP-LF was used for 0h/Untreated control, resulting in partial overlap between the images. Figure 7A has now been updated to include the correct data for 0h/Untreated control sample.

The original Figure 5 and Figure 7 and accompanying legends appear below.

**Fig. 5:**
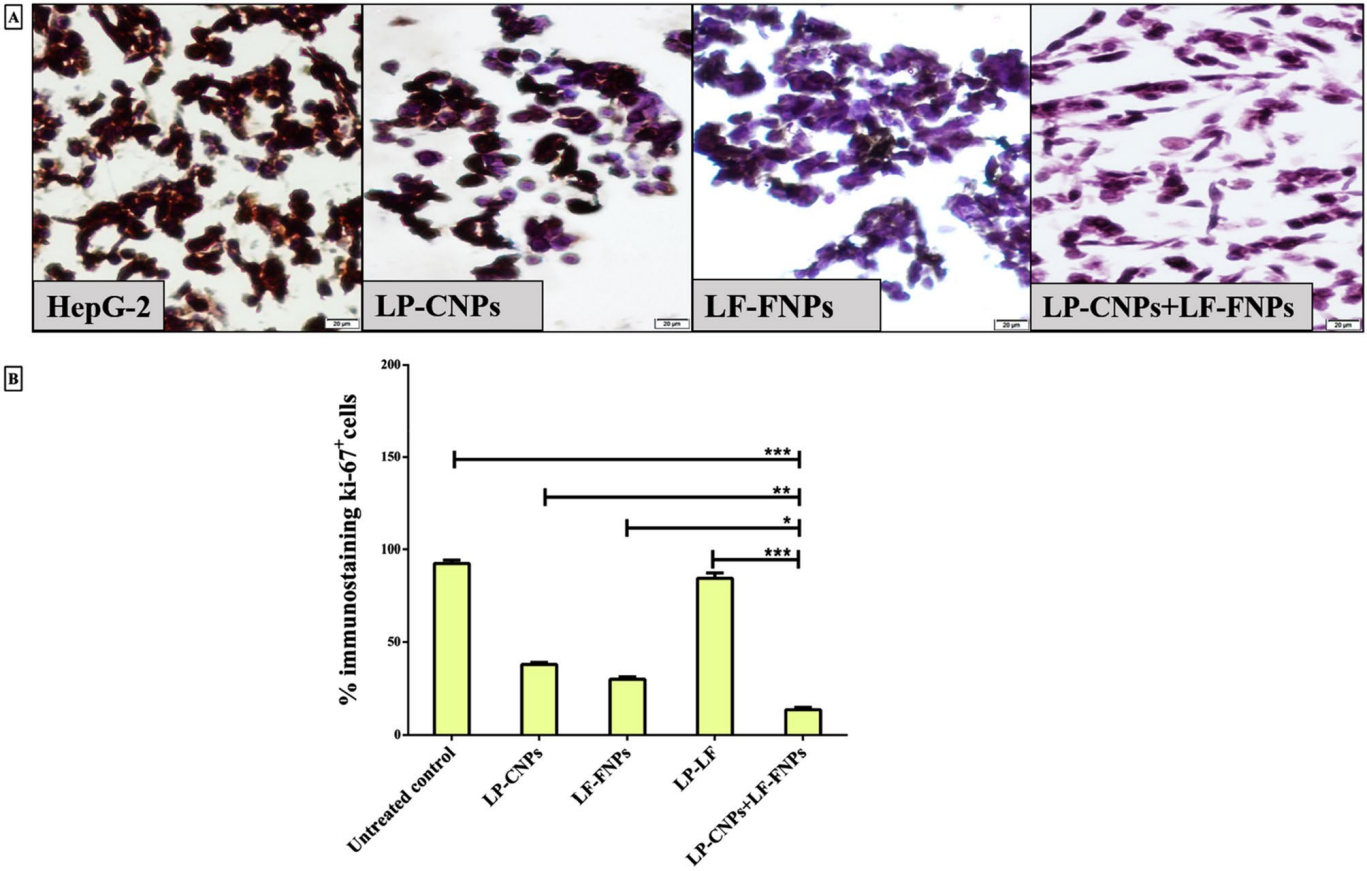
Immunohistochemical analysis of proliferation marker (Ki-67) in HepG2 cells after treatment with the most effective anticancer nanoformulas and LP + LF. (**A**) Immunohistochemical staining images of the untreated and LP-CNPs, LF-FNPs, LF-LP and LP-CNPs + LF-FNPs -treated HepG2 with (**B**) Percentage of Ki-67^+^-immunostained cell population in the untreated and treated HepG2 cells (Magnification 200 X; scale bar 20 µm). All values are demonstrated as mean ± S.E. LP-CNPs + LF-FNPs are statistically significant with other formulas at p < 0.05*, p < 0.005**, p < 0.0005***. CNPs: CuO nanoparticles; FNPs: iron nanoparticles; LF: lactoferrin; LP: lactoperoxidase.

**Fig. 7:**
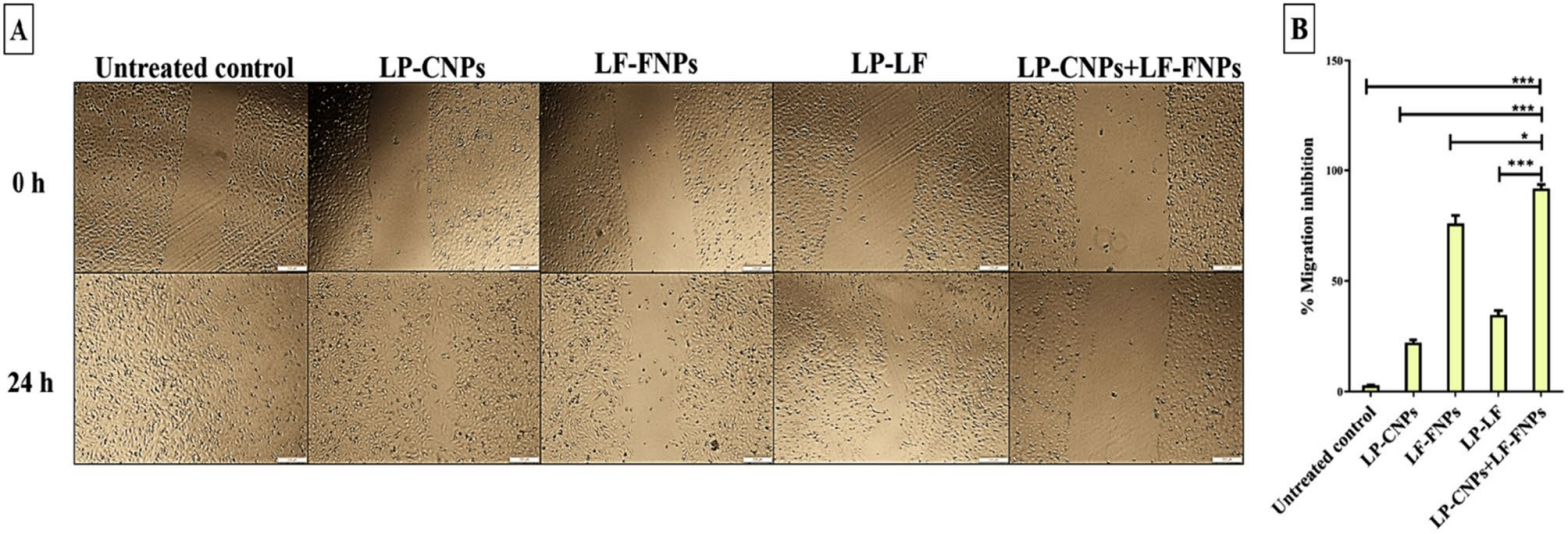
Anti-migration potency using wound healing assay. (A) Microscopic image of the scratched wound area in the untreated and LP-CNPs, LF-FNPs, LF-LP and LP-CNPs + LF-FNPs -treated Caco2 cells at 0 and 24 h (Magnification × 40; scale bar 200 µm); (B) relative migration inhibition percentages. All values are demonstrated as mean ± SE. LP-CNPs + LF-FNPs are statistically significant with other formulas at p < 0.05*, p < 0.005**, p < 0.0005***. CNPs: CuO nanoparticles; FNPs: iron nanoparticles; LF: lactoferrin; LP: lactoperoxidase.

The original Article has been corrected.
