# Supplementary material for: Augmenting apoptosis-mediated anticancer activity of lactoperoxidase and lactoferrin by nanocombination with copper and iron hybrid nanometals
Source: Sci Rep. 2022 Aug 1;12:13153. doi: 10.1038/s41598-022-17357-y (PMC9343395; doi:10.1038/s41598-022-17357-y)
Supplement: Supplementary file 1 — Supplementary Information. [file 41598_2022_17357_MOESM1_ESM.docx]

**
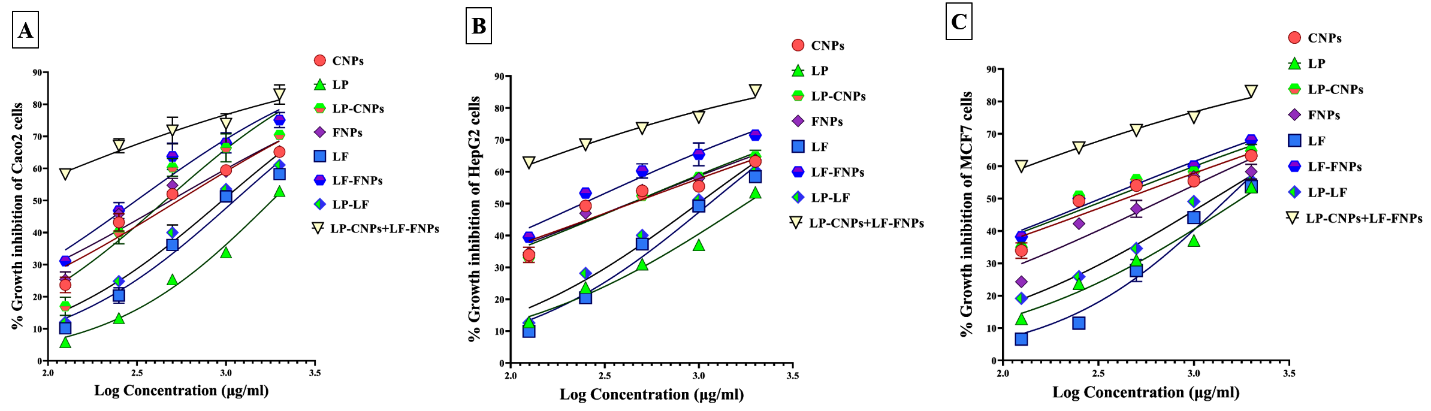
**

**Figure S1.** Dose response curve of all prepared formulas for inhibiting growth of Caco2, HepG2 and MCF7 cells after 72 h incubation. All values are demonstrated as mean±SEM.

**Table S1**. CT values of gene expression p53 and BCl2 in MCF7 cells treated with LP-CNPs, LF-FNPs, LF-LP or LP-CNPs+LF-FNPs.

| Sample | p53 | | | Housekeeping gene | | | BCl2 | | |
| --- | --- | --- | --- | --- | --- | --- | --- | --- | --- |
| Untreated | 36.23 | 34.54 | 35.26 | 25.12 | 25.62 | 27.66 | 14.00 | 12.29 | 10.23 |
| LP-CNPs | 32.91 | 32.93 | 32.77 | 24.36 | 25.56 | 25.67 | 11.68 | 11.57 | 11.72 |
| LF-FNPs | 32.94 | 32.71 | 32.90 | 26.00 | 26.11 | 24.67 | 12.79 | 12.63 | 12.26 |
| LP-LF | 34.25 | 34.12 | 34.42 | 26.78 | 26.09 | 24.98 | 12.32 | 12.08 | 12.05 |
| LP-CNPs+LF-FNPs | 31.70 | 31.18 | 31.76 | 26.65 | 24.94 | 25.05 | 14.77 | 14.63 | 14.85 |
